# Chronic ascites as the initial presentation of systemic lupus erythematosus in a 37-year-old Syrian female patient: a case report

**DOI:** 10.1186/s13256-026-05840-3

**Published:** 2026-03-03

**Authors:** Ahmad Muhammad, Bashar Badawi, Kawthar AlKhellow, Rama Abdulaziz, Omar M. S. Farhoud

**Affiliations:** 1 Faculty of Medicine, University of Aleppo, Aleppo, Syria; 2 Department of Gastroenterology, Aleppo Hospital for Internal Diseases, Aleppo, Syria; 3 Department of Rheumatology, Aleppo Hospital for Internal Diseases, Aleppo, Syria

**Keywords:** Systemic lupus erythematosus, Chronic ascites, Autoimmune disease, Lupus peritonitis, Deep venous thrombosis, Case report

## Abstract

**Background:**

Systemic lupus erythematosus is a chronic autoimmune disease characterized by immune complex deposition in various organs and autoantibodies against nuclear antigens. Gastrointestinal involvement is a common complication in systemic lupus erythematosus, affecting many patients, often asymptomatically. While serositis is a common manifestation of systemic lupus erythematosus, peritoneal involvement, particularly as the initial symptom, is rare.

**Case presentation:**

A 37-year-old Syrian housewife presented with chronic painless abdominal distension, pain in the left leg accompanied by non-pitting edema, and a productive cough. Initial evaluation revealed right pleural effusion on chest imaging and ascites on abdominal ultrasound and computed tomography. Paracentesis demonstrated yellowish, predominantly lymphocytic fluid with a low serum ascites albumin gradient [1] and low total protein (1.71 g/dL), while peritoneal biopsy indicated mild chronic nonspecific peritonitis. Routine laboratory tests, including liver function and 24-hour urine protein, were unremarkable. Given the clinical picture and serological findings, specifically a positive anti-double-stranded DNA antibody in the context of pleural effusion, the diagnosis of systemic lupus erythematosus was established on the basis of the American College of Rheumatology criteria. The patient was treated with hydroxychloroquine (200 mg/day) and low-dose prednisolone (5 mg/day), resulting in significant clinical improvement with resolution of ascites, while her leg symptoms improved with apixaban (10 mg/day) and enoxaparin (60 mg/day for 2 days) use. On follow-up, full recovery was achieved, and the patient remained asymptomatic.

**Conclusion:**

This case underscores the atypical and challenging presentation of systemic lupus erythematosus, emphasizing the necessity of considering a broad spectrum of potential diagnoses in cases of unexplained ascites, particularly recognizing systemic lupus erythematosus as a diagnosis of exclusion.

**Supplementary Information:**

The online version contains supplementary material available at 10.1186/s13256-026-05840-3.

## Background

Systemic lupus erythematosus (SLE) is a chronic multisystem autoimmune illness. SLE is defined by the deposition of immune complexes in several organs and autoantibodies against nuclear antigens. SLE can present with a wide variety of potentially dangerous symptoms [1]. The most common findings are hematological abnormalities, skin, renal, and central nervous system involvement [2]. The prevalence of SLE is significantly higher in female individuals, being ten times more common than in male individuals, with the highest occurrence observed in individuals between the ages of 25 and 44 years [1].

In 40–60% of patients with SLE, gastrointestinal involvement is a common complaint. In 8–10% of individuals, gastrointestinal signs that are clinically recognizable have been reported. However, autopsy reports reveal gastrointestinal involvement in 60–70% of individuals, indicating a typical occurrence of asymptomatic or undetected involvement [2].

Among the many different signs that patients with SLE can experience, serositis is frequently found. Pleuritis and/or pericarditis affect about 16% of patients with SLE. Further, peritoneal involvement is rare, and SLE presenting with ascites as the initial symptom is even rarer [1].

In this report, the patient was diagnosed with systemic lupus erythematosus with the first manifestation being chronic ascites.

## Case presentation

A 37-year-old Syrian housewife presented complaining of severe painless abdominal distension that had been developing for a year, and pain in the left leg accompanied by non-pitting edema developed rapidly 2 days before attending the hospital. She also mentioned a productive cough with white sputum. The patient was admitted to another hospital 20 days previously for the abdominal distension before she was referred to our hospital. At admission, the patient had a loss of appetite, insomnia, and irregular menstruation. The ascites had interfered with her ability to perform daily household activities. She denied smoking, alcohol use, or any familial history of autoimmune diseases or connective tissue disorders. She had an appendectomy 15 years ago.

On physical examination, the patient was acyanotic, and the general state was bad. Her blood pressure was 110/70 mmHg, SpO_2_ 99%, heart rate 114 beats per minute, Glasgow coma scale 15/15, and pupils were reactive. Cardiac system exam showed normal. Neurological examination was unremarkable. Chest examination showed weakened respiratory sounds at the base of the right lung, which suggested right pleural effusion, and the patient experienced first-grade dyspnea. On abdominal examination, there was abdominal distension without tenderness or abdominal defense reflex, and there were no palpable masses or hepatic or splenic enlargement. There was non-pitting edema in the left leg, thus deep venous thrombosis (DVT) was suspected. The presentation timeline is summarized in Table 4.

The laboratory test results are summarized in Table 1, while liver function tests results are presented in Table 2. C-reactive protein (CRP) was 17.6 mg/L, hepatitis B virus (HBV) and hepatitis C virus (HCV) tests were negative, thyroid-stimulating hormone (TSH) level was 3.4 μIU/mL (within the normal range of 0.3–4.0 μIU/mL), and the 24-hour urine protein and volume were normal.

**Table 1:** Laboratory test results

HGB	13.5	12–16 g	Glucose	67	70–105 mg/dL
PLT	195	150–450	Urea	16	15–45 mg/dL
WBCs	10,000	4000–11,000	Creatinine	0.47	Woman 0.6–1.1 mg/dL
					Man 0.7–1.3 mg/dL
Neu	79.1	55–70%	LDH	6222	480 UI/L
Lym	15.7	20–45%

**Table 2:** Liver function tests

PT	15.4	14 s	Total protein	5.7	6–8 g/L
Prothrombin activity	87	75–100%	Albumin	3.86	3–5 g/L
INR	1.1	1.2–1.5	Total bilirubin	0.51	0.2–1.1 mg/dL
ALT	24	Up to 45 UI/L	Conjugated bilirubin	0.08	0.08–0.35 mg/dL
AST	15	Up to 40 UI/L	Unconjugated bilirubin	0.43	0.2–0.8 mg/dL

**Fig. 1:**
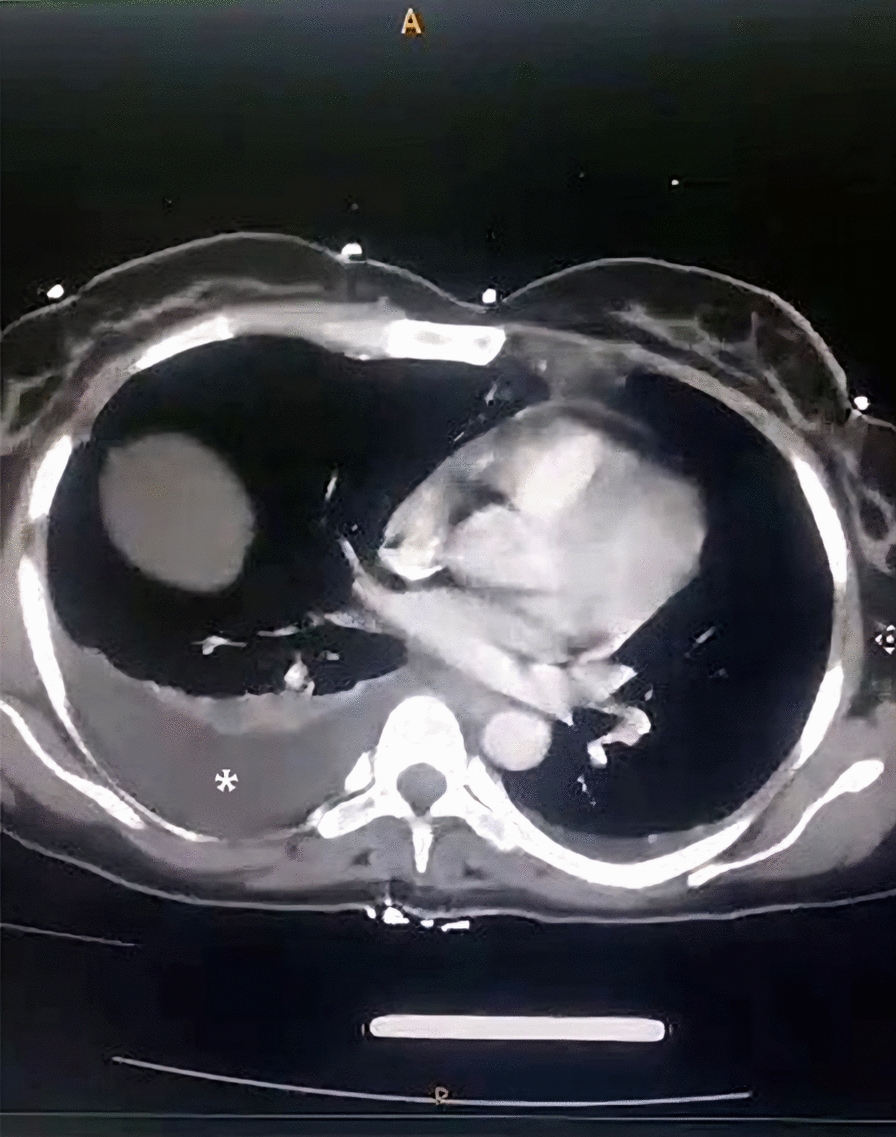
Axial computed tomography section of the chest showing large pleural effusion (white asterisks)

**Fig. 2:**
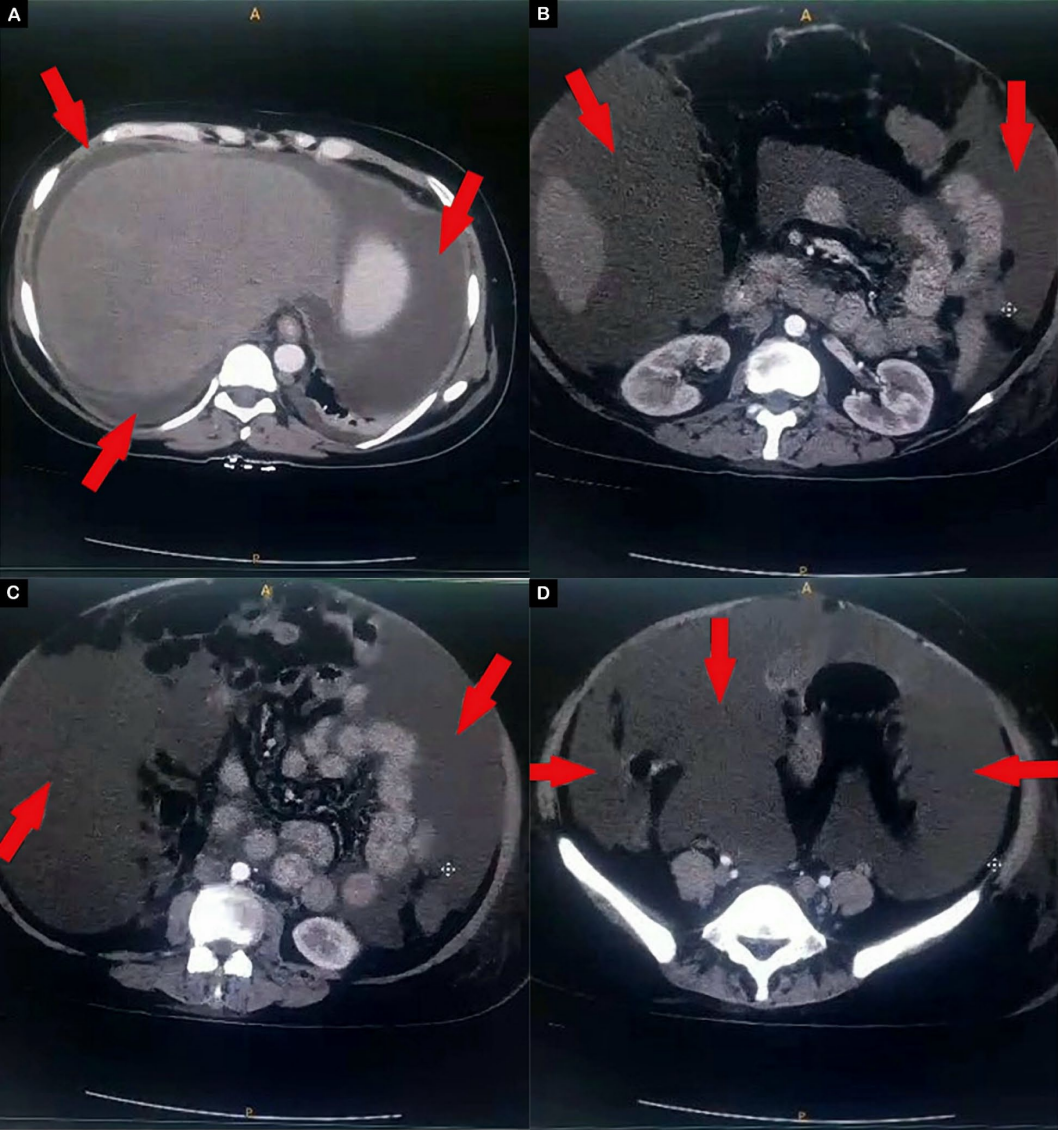
Axial computed tomography section of the abdomen showing massive ascites (red arrows). **A** At the T9–T10 level, at the superior aspect of the liver adjacent to the diaphragmatic, dome fluid can be observed, consistent with a subphrenic fluid collection. **B** At the T12–L1 level, free fluid is present between the bowel loops. **C** At the L1–L2 vertebral level. **D** At the L3–L4 vertebral level, at the region of the iliac wing

**Fig. 3:**
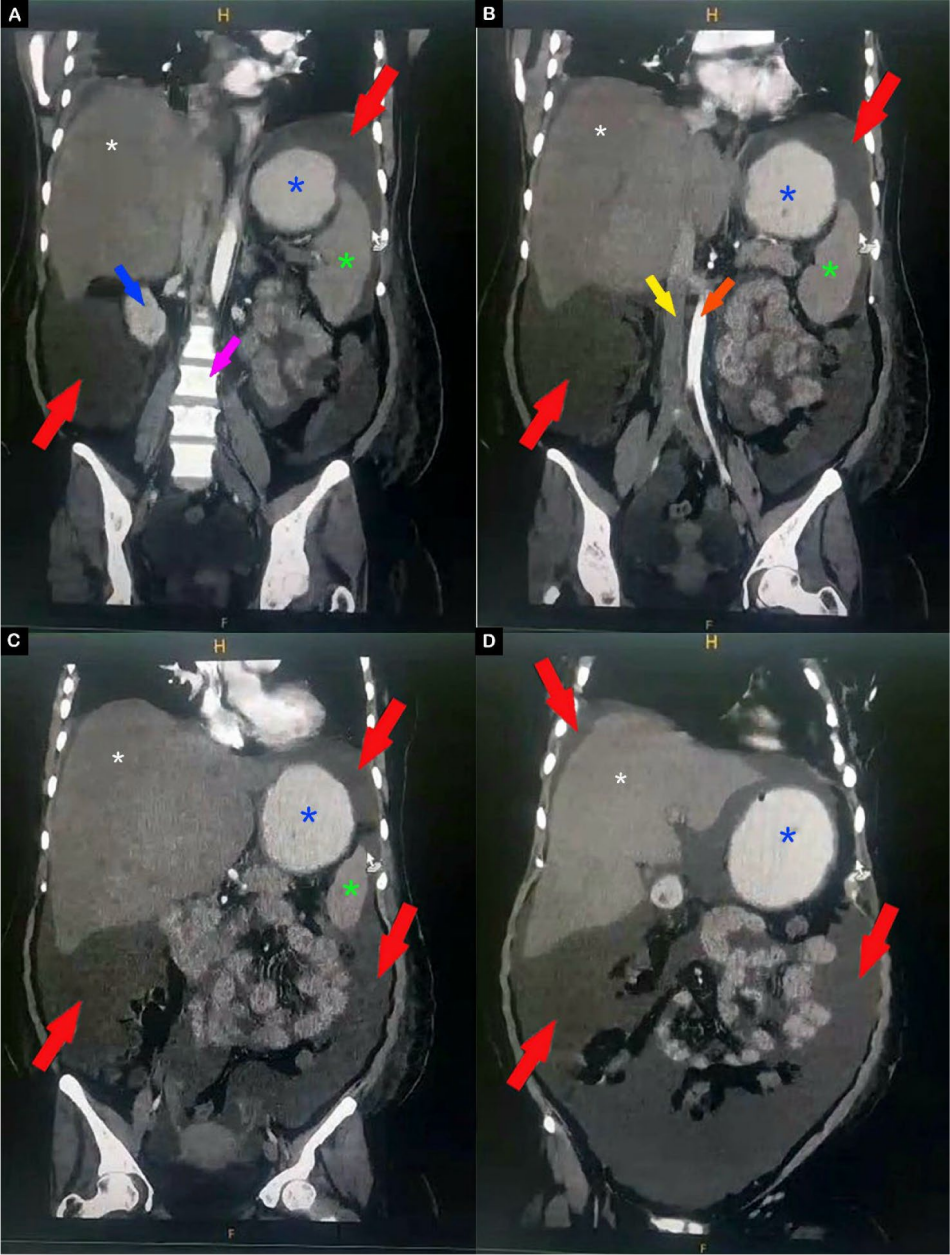
Coronal computed tomography sections of the abdomen showing massive ascites (red arrows). **A** Posterior coronal section showing the right kidney (blue arrow), lumbar vertebrae (pink arrow), liver (white asterisk), stomach (blue asterisk), and spleen (green asterisk). **B** Mid-abdomen coronal section displaying the inferior vena cava (yellow arrow) and the abdominal aorta (orange arrow). **C** Mid-abdomen coronal section demonstrating massive ascites surrounding the bowel loops (red arrows). **D** Anterior coronal section revealing a subphrenic fluid collection above the liver

Chest X-ray demonstrated a right-sided pleural effusion and barrel chest, confirmed by computed tomography (CT) imaging (Fig. 1). The abdominal ultrasound showed ascites without any other abnormalities, and the abdominal CT confirmed the findings (Figs. 2, 3). Venous Doppler ultrasound revealed DVT in the left deep femoral vein. Echocardiogram was normal, ruling out cardiac failure.

Puncture of the ascitic fluid showed yellowish fluid with the following findings: white blood cell (WBC) count was 120/mm^3^, while red blood cell (RBC) count was 100/mm^3^. The differential count for neutrophils and lymphocytes was 4% and 96%, respectively, and serum ascites albumin gradient (SAAG) was 1. The total protein was 1.71 g/dL, and glucose 123 mg/dL. Adenosine deaminase was 7.4 (normal up to 33 U/L). Ascitic fluid pathology showed noninflammatory, nontumoral fluid with few lymphoid and mesothelial cells. Peritoneal biopsy reported mild chronic nonspecific peritonitis.

On the basis of the clinical findings and investigations, several common causes of ascites were excluded. The normal liver functions, abdominal ultrasound, and negative viral hepatitis tests excluded hepatic cirrhosis, hepatitis-related ascites, and hepatic outflow obstruction. Ascetic fluid analysis and peritoneal biopsy showed no evidence for peritoneal malignancy or peritoneal tuberculosis. The normal renal function and 24-hour urine protein ruled out nephrotic syndrome. The absence of pancreatic abnormalities on contrast-enhanced CT, together with the lack of typical abdominal pain, made pancreatitis improbable. These findings collectively raised the suspicion of a rheumatologic cause, thus we performed rheumatoid arthritis quantitative, ANA8-screen, and double-stranded (ds)DNA-G tests. The results are presented in Table 3.

**Table 3:** Autoimmune and inflammation-related tests

ESR (1 hour)	30	Up to 7 mm	ANA 8-screen	2.2	Up to 1.2 index
ESR (2 hours)	61	Up to 20 mm	dsDNA-G	24.3	Up to 18 IU/mL
RA test	12.8	Up to 14 IU/mL			

On the basis of the 2019 European League Against Rheumatism/American College of Rheumatology Classification Criteria for Systemic Lupus Erythematosus, systemic lupus erythematosus was suspected: anti-dsDNA antibody and pleural or pericardial effusion [3]. Hydroxychloroquine (Plaquenil 200 mg/day) and prednisolone 5 mg/day were administered. Additionally, apixaban (10 mg/day) and enoxaparin (60 mg/day for 2 days) were administrated for the DVT. The patient showed improvement in her general condition and reduction in abdominal circumference and started outpatient follow-up. On follow-up, the patient has recovered well. She is currently being followed up at the outpatient clinic and remains asymptomatic, with no ascites (Table 4).

**Table 4:** Presentation timeline during hospitalization

Day since admission	Signs and symptoms
Day 1	Severe painless abdominal distension
	Pain in the left leg accompanied by non-pitting edema
	Productive cough
Day 2	Weakened respiratory sound at the base of the right lung
	Leg pain
	Abdominal pain
	Fever
Day 5	Exertional dyspnea
Day 9	Fever and night sweats
Day 11	Nonproductive cough

## Discussion

SLE is a multifactorial autoimmune disorder with systemic implications, often affecting the gastrointestinal system. Despite its often-silent nature, SLE can manifest with diverse gastrointestinal complications, including mesenteric vasculitis, protein-losing enteropathy, intestinal pseudo-obstruction, and acute pancreatitis. Furthermore, SLE may be associated with other conditions such as celiac disease and inflammatory bowel disease [1].

Gastrointestinal manifestations in SLE are characterized by varied and nonspecific presentation, encompassing symptoms such as nausea, vomiting, diarrhea, malabsorption, acute abdominal pain, and ascites [2]. The presence of these symptoms is indicative of a variety of underlying conditions, making it challenging to determine whether they are secondary manifestations of systemic lupus erythematosus (SLE) when they are the only existing symptoms [1].

In SLE, pleuritis and pericarditis are the major findings, while ascites is a rare manifestation of lupus serositis, especially massive ascites [4, 5]. It is generally associated with active disease or occurs due to nephrotic syndrome, protein-losing enteropathy, constrictive pericarditis, and conditions disassociated with lupus. It is exceedingly rare if peritonitis occurs as the first presenting manifestation of SLE and is unrelated to the conditions mentioned earlier [6].

Ascites can be acute or chronic, and present with different symptoms such as abdominal distension, discomfort, or pain. Additionally, marked ascites has been linked to chronic lupus peritonitis, and is assumed to be disassociated from disease activity [5].

This case is extremely rare, showcasing massive chronic ascites that had developed for a year as the initial presentation without the typical manifestations of SLE. Common causes of ascites, such as malignancies, tuberculosis, and other causes mentioned previously, were ruled out, leading to the suspicion of an autoimmune disorder. Subsequent serology tests, including antinuclear antibody (ANA) and anti-dsDNA, supported a diagnosis of SLE. Despite the absence of typical symptoms of SLE as defined by the American College for Rheumatology [3], with a total score of 11 points, the patient met the 10-point threshold needed to be diagnosed with SLE. The patient’s full recovery on the follow-up confirmed the diagnosis.

Massive chronic ascites as the initial presentation of SLE can pose a real challenge for the physician without the typical manifestations of SLE. Confirming the need for comprehensive evaluation when faced with unexplained ascites and the absence of typical manifestations of SLE does not rule out the diagnosis of SLE.

Precise identification of ascites etiology is crucial for treatment. Portal hypertension and peritoneal disease contribute to abdominal fluid buildup through mechanisms such as sodium and water retention, increased plasma volume, and serosal fluid secretion. These processes are mediated by the renin–angiotensin–aldosterone system. Fluid analysis is the initial step to identify the etiology. In portal hypertension, the fluid is transudative because it is caused by vascular hydrostatic pressure, whereas in inflammatory or neoplastic peritoneal disease, the vascular permeability changes, leading to the production of protein-rich exudative fluid. The ascites development mechanism in lupus peritonitis is inadequately understood. However, two theories explain it. The first posits that production of autoantibodies by B-lymphocytes is the mechanism. The precipitation of these antibodies on the peritoneum, binding to antigens and forming immune complexes, causes a local inflammatory response. The second one assumes that the inflammation of peritoneal vessels or serous membranes covering abdominal organs causes the ascites. As a result, the ascites fluid is exudative because of peritoneal involvement in both circumstances [5].

In lupus peritonitis, ascitic fluid characteristics involve a serum ascites albumin gradient (SAAG) of less than 1.1. It can involve a wide range of WBCs, from 10 to 1630/mm^3^, and a range of fluid protein, from 34 mg/L to 47 mg/L [1].

Diagnosing lupus peritonitis requires ruling out the most common causes of exudative ascites, such as peritoneal carcinomatosis, primary mesothelioma, peritoneal pseudomyxoma, hepatocellular carcinoma, peritoneal tuberculosis, nephrotic syndrome, protein-losing enteropathy, and severe malnutrition [1, 7]. Computed tomography was the most often conducted exam, followed by radiography, ultrasound, and colonoscopy [2].

The standard treatment for this condition typically involves using nonsteroidal antiinflammatory drugs and corticosteroids. Despite successful treatment in many cases, refractory SLE does occur. When standard treatments fail, immunomodulatory or immunosuppressive therapies may be used. In some situations, surgical interventions may also be considered [1].

Immune system disorders may raise the risk of thrombotic events such as deep vein thrombosis (DVT). These findings suggest a possible link between immune system disorders and DVT. SLE remains a significant risk factor for thrombosis, even if the risk declines after the first year of follow-up. Antimalarial drugs and aspirin are used in prevention [8].

## Conclusion

Although serositis is frequently observed in SLE, peritoneal involvement is very rare, especially as the disease’s initial manifestation. This case emphasizes that even in the absence of the typical causes mentioned in SLE diagnosis criteria, SLE should be considered as a differential diagnosis of exudative ascites, taking it into account after excluding common causes such as nephrotic syndrome, protein-losing enteropathy, and constrictive pericarditis. It also highlights the need for comprehensive evaluation when faced with unexplained ascites.

## Supplementary Information

Additional file 1.

## Data Availability

Not applicable.

## Abbreviations

SLE: Systemic lupus erythematosus
SpO_2_: Peripheral capillary oxygen saturation
BPM: Beats per minute
CRP: C-reactive protein
HBV: Hepatitis B virus
HCV: Hepatitis C virus
TSH: Thyroid-stimulating hormone
DVT: Deep venous thrombosis
SAAG: Serum ascites albumin gradient
WBC: White blood cell count
RBC: Red blood cell count
ANA: Antinuclear antibody
dsDNA: Double-stranded DNA
