# Supplementary material for: Chronic ascites as the initial presentation of systemic lupus erythematosus in a 37-year-old Syrian female patient: a case report
Source: J Med Case Rep. 2026 Mar 3;20:173. doi: 10.1186/s13256-026-05840-3 (PMC13064269; doi:10.1186/s13256-026-05840-3)
Supplement: Supplementary file 1 — Additional file 1. [file 13256_2026_5840_MOESM1_ESM.docx]

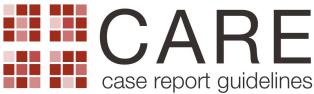
**CARE Checklist (2013) of information to include when writing a case report
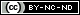
**

**Topic Item Checklist item description Reported on Page**

**Title 1** The words “case report” should be in the title along with the area of focus 1

**Key Words 2** 2 to 5 key words that identify areas covered in this case report 2

**Abstract 3a** Introduction—What is unique about this case? What does it add to the medical literature? 2

**3b** The main symptoms of the patient and the important clinical findings 2

**3c** The main diagnoses, therapeutics interventions, and outcomes 2

**3d** Conclusion—What are the main “take-away” lessons from this case? 2

**Introduction 4** One or two paragraphs summarizing why this case is unique with references 2

**Patient Information 5a** Demographic information and other patient specific information 3

**5b** Main concerns and symptoms of the patient 3

**5c** Medical, family, and psychosocial history including relevant genetic information (also see timeline). 3

**5d** Relevant past interventions and their outcomes N/A

**Clinical Findings 6** Describe the relevant physical examination (PE) and other significant clinical findings 3

**Timeline 7** Important information from the patient’s history organized as a timeline 3

# Diagnostic Assessment

**Therapeutic Intervention**

**Follow-up and Outcomes**

**8a** Diagnostic methods (such as PE, laboratory testing, imaging, surveys) 3

**8b** Diagnostic challenges (such as access, financial, or cultural) N/A

**8c** Diagnostic reasoning including other diagnoses considered 3

**8d** Prognostic characteristics (such as staging in oncology) where applicable N/A

**9a** Types of intervention (such as pharmacologic, surgical, preventive, self-care) 3

**9b** Administration of intervention (such as dosage, strength, duration) 3

**9c** Changes in intervention (with rationale) 3

**10a** Clinician and patient-assessed outcomes (when appropriate) 3

**10b** Important follow-up diagnostic and other test results 3

**10c** Intervention adherence and tolerability (How was this assessed?) N/A

**10d** Adverse and unanticipated events N/A

**Discussion 11a** Discussion of the strengths and limitations in your approach to this case 3

**11b** Discussion of the relevant medical literature 3, 4

**11c** The rationale for conclusions (including assessment of possible causes) 4

**11d** The primary “take-away” lessons of this case report 5

**Patient Perspective 12** When appropriate the patient should share their perspective on the treatments they received N/A

**Informed Consent 13** Did the patient give informed consent? Please provide if requested . . . . . . . . . . . . . . . . . . . . . . . . . . . . . . . . . . . . . .**Yes**
